# The effects of light exposure during incubation on embryonic development and hatchling traits in lizards

**DOI:** 10.1038/srep38527

**Published:** 2016-12-05

**Authors:** Yong-Pu Zhang, Shu-Ran Li, Jun Ping, Shi-Wen Li, Hua-Bin Zhou, Bao-Jun Sun, Wei-Guo Du

**Affiliations:** 1 College of Life and Environmental Science, Wenzhou University, 325035, Wenzhou, Zhejiang, People’s Republic of China; 2 Key Laboratory of Animal Ecology and Conservation Biology, Institute of Zoology, Chinese Academy of Sciences, Beijing 100101, People’s Republic of China

## Abstract

Light is an environmental factor that is known to profoundly affect embryonic development in some oviparous vertebrates, but such effects are unstudied in reptiles. We investigated the light sensitivity of lizard embryos by examining the thickness and light transmittance of eggshells as well as the effect of light on embryonic development and hatchling traits in four lizard species, the Chinese skink (*Plestiodon chinensis*), the northern grass lizard (*Takydromus septentrionalis*), the oriental leaf-toed gecko (*Hemidactylus bowringii*) and the Japanese gecko (*Gekko japonicus*). The eggshells were thinner and thus had higher light transmittance in Chinese skink than the other three species. Light exposure during incubation significantly accelerated the embryonic development in all species, with higher light intensity resulting in faster embryonic development. Interestingly, light stimulation negatively influenced hatchling size and survival in skinks, but had no effect in lacertids and geckos. This interspecific discrepancy not only relates to the differences in thickness and light transmittance of eggshells, but might also reflect the differences in the reproductive habits of these species. Given the diversity of light conditions that reptile embryos face during development, studies on the response of reptile embryos to light may offer a unique opportunity to understand the mechanisms of embryonic light sensitivity in animals.

Light plays an important role in the development and growth of organisms^1^,^2^. In many oviparous animals, light is essential for the embryonic development, and dark conditions may result in hatching failure or malformation of hatchlings^3^,^4^. Light stimulation can also accelerate embryonic development, largely because light stimulation can increase embryonic metabolism^2^,^4^,^5^,^6^. In addition, exposure to light during embryonic development strongly affects offspring phenotypes such as patterns of lateralization in fish and birds^7^,^8^.

Despite the importance of light for embryonic development in other oviparous animals, ecologists have paid little attention to these effects in reptiles. It has traditionally been assumed that light is not an important influence on embryonic development in reptiles because most oviparous reptiles bury their eggs in the ground, thus sheltering them from light. However, this assumption is not valid for all oviparous reptiles, which exhibit diverse array of life history traits and reproductive strategies. In fact, light likely affects embryonic development and offspring fitness in some lizard species (e.g., geckos), which often lay their eggs in leaf litter, rock crevices, or tree holes, where embryos are potentially exposed to light^9^,^10^,^11^. These varying incubation conditions raise the question whether light affects embryonic development and offspring traits in lizards.

Because reptiles represent a transition from anamniota to amniote in the evolutionary history of vertebrates, determining whether and how lizard embryos respond to light will shed light on the underlying mechanisms and adaptive evolution of light sensitivity in animals. To reveal whether light affects embryonic development and offspring traits in lizards, we measured the thickness of eggshells, and investigated the effects of light on embryonic development and hatchling phenotypes in four lizard species, one skink, one lacertid and two geckos. The four lizard species produce eggs with different thickness of eggshells^10^. We hypothesize that the effects of light on embryonic development and hatchling traits would be negatively related to the thickness of eggshell, if thick eggshells reduce light transmittance and therefore protect the embryos from light damage.

## Results

**Figure 1:**
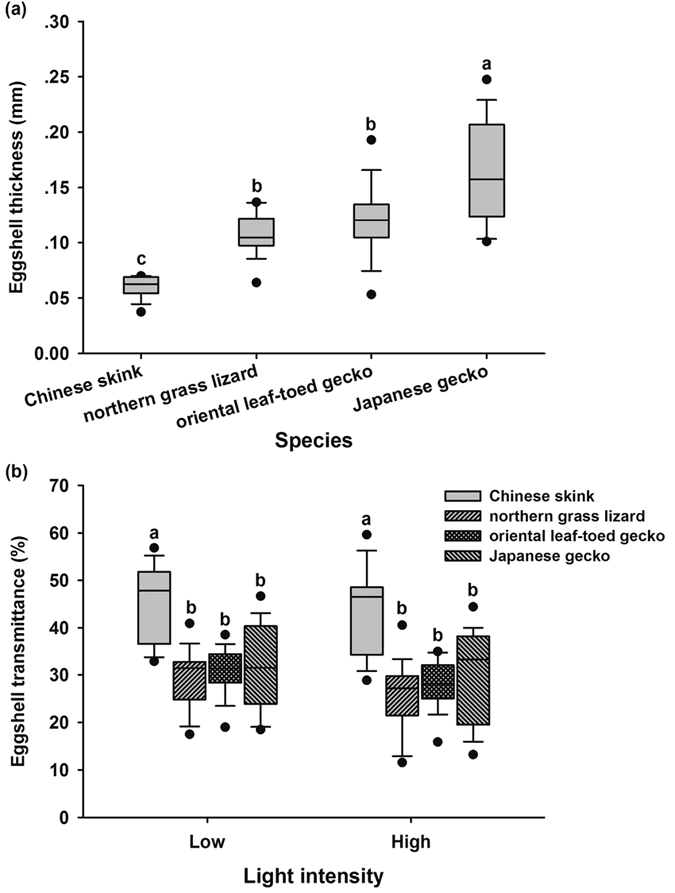
The thickness (**a**) and light transmittance (**b**) of eggshell in the four lizard species, the Chinese skink (n = 17), the northern grass lizard (n = 17), the oriental leaf-toed gecko (n = 16) and the Japanese gecko (n = 21). The line in the box is the median. Whiskers represent the 10th and 90th percentiles. Black circles beyond the boxplots show the 5th to 95th percentiles. Different alphabets above the boxes indicate significant difference (Tukey’s post-hoc test).

The thickness of eggshells differed between species (*F*_3, 67_ = 33.39, *P* < 0.001), with the Chinese skink (*Plestiodon chinensis*) having the thinnest eggshell, the Japanese gecko (*Gekko japonicus*) the thickest eggshell, and the northern grass lizard (*Takydromus septentrionalis*) and the oriental leaf-toed gecko (*Hemidactylus bowringii*) in between (Fig. 1a). The light transmittance of eggshell was higher in the Chinese skink than other three species (*F*_3, 67_ = 18.61, *P* < 0.001) (Fig. 1b).

**Figure 2:**
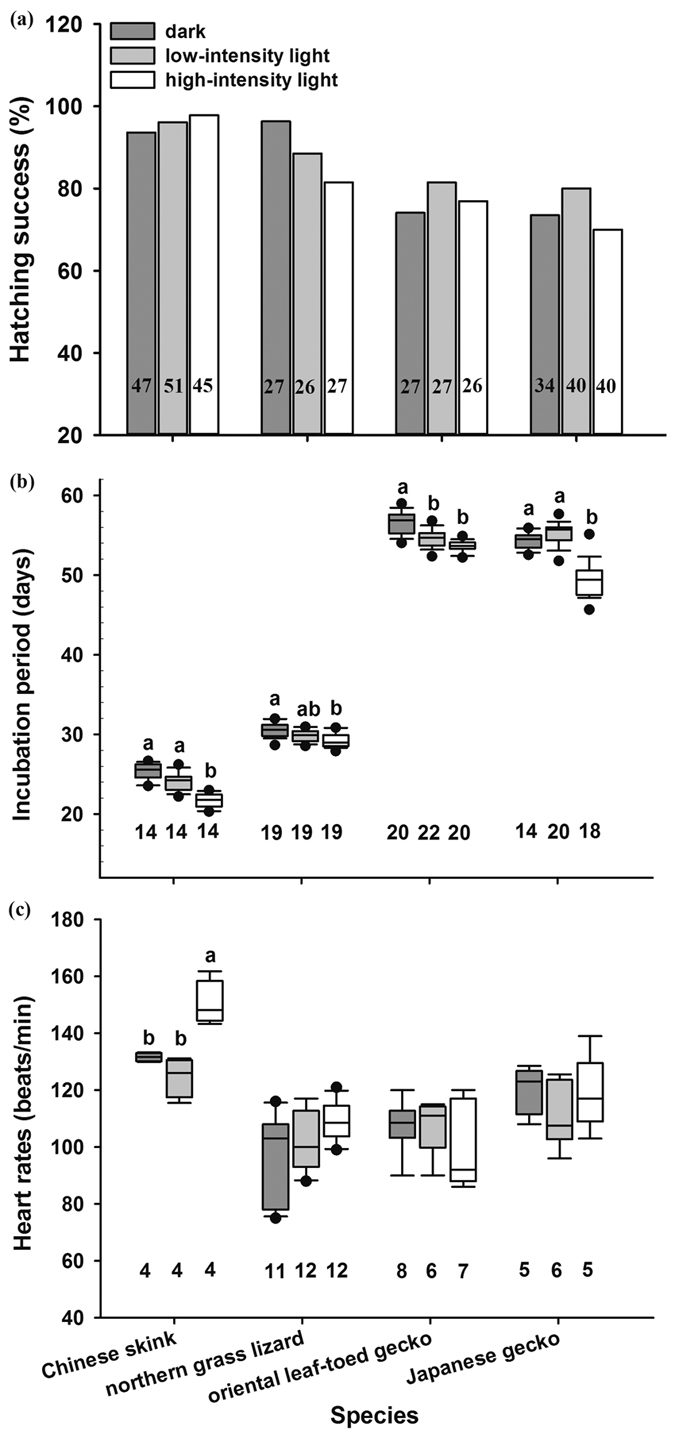
Hatching success (**a**), incubation period (**b**) and heart rate (**c**) of embryos in the Chinese skink, the northern grass lizard, the oriental leaf-toed gecko and the Japanese gecko incubated under dark and light conditions. The line in the box is the median. Whiskers represent the 10th and 90th percentiles. Black circles beyond the boxplots show the 5th to 95th percentiles. Different alphabets above the boxes indicate significant difference (Tukey’s post-hoc test). Sample sizes are shown on the columns or below the boxplots.

**Figure 3:**
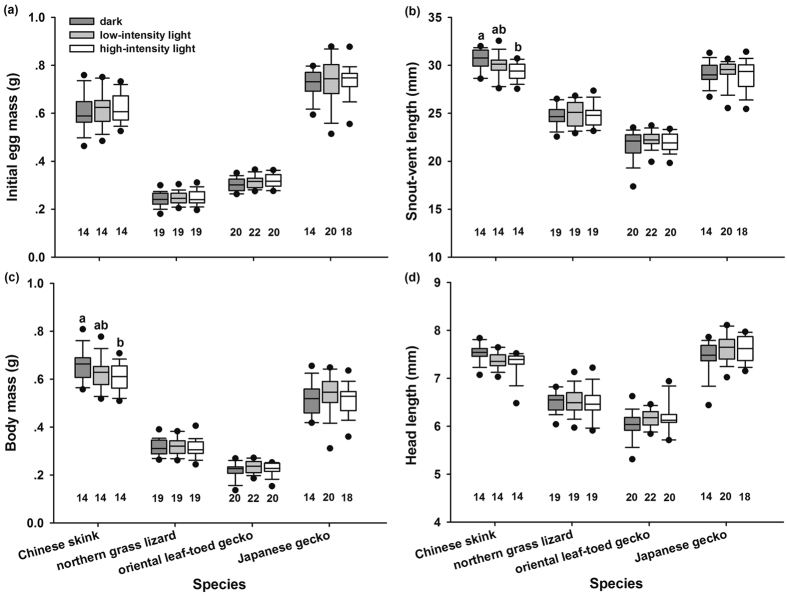
Initial egg mass (**a**) and hatchling morphology (**b**) snout-vent length; (**c**) body mass; (**d**) head length) in the Chinese skink, the northern grass lizard, the oriental leaf-toed gecko and the Japanese gecko incubated under dark and light conditions. The line in the box is the median. Whiskers represent the 10th and 90th percentiles. Black circles beyond the boxplots show the 5th to 95th percentiles. Different alphabets above the boxes indicate significant difference (Tukey’s post-hoc test). Sample sizes are shown below the boxplots.

**Figure 4:**
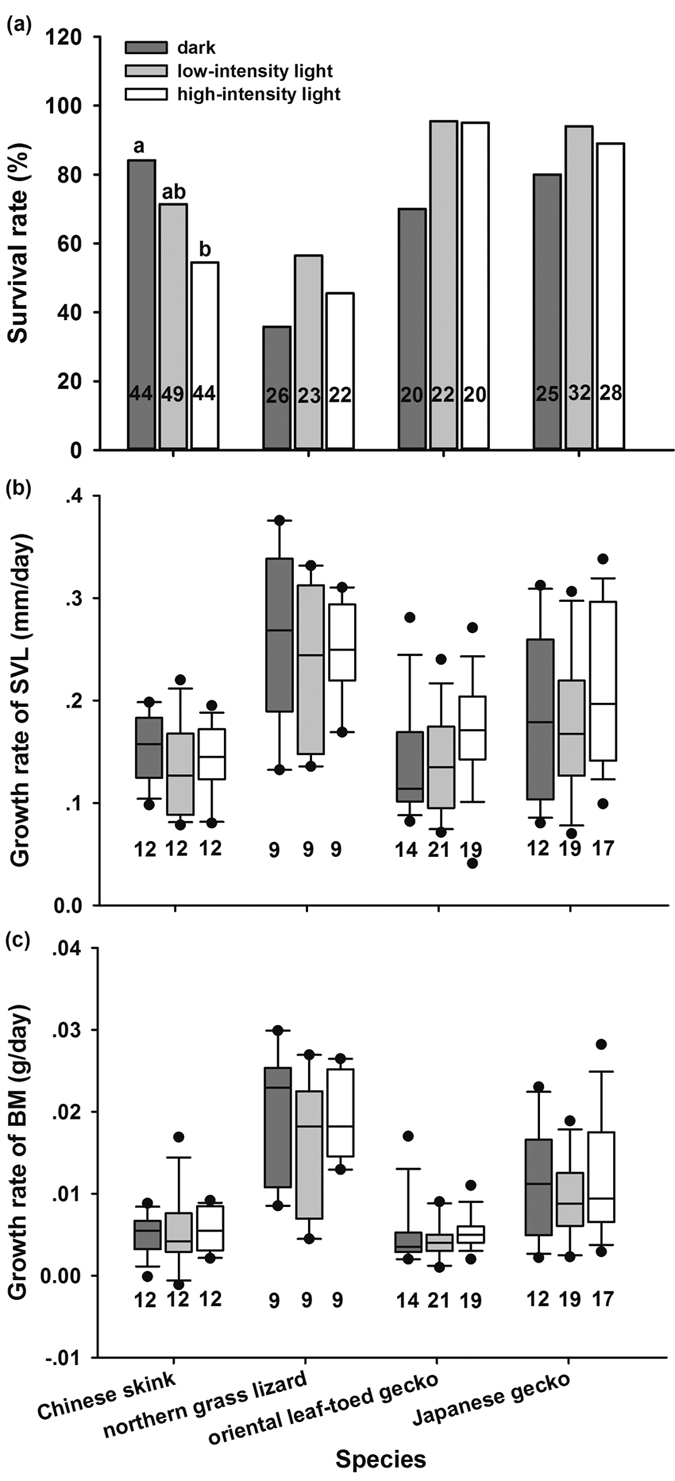
Hatchling survival rate (**a**), growth rate of snout-vent length (SVL) (**b**) and body mass (BM) (**c**) in the Chinese skink, the northern grass lizard, the oriental leaf-toed gecko and the Japanese gecko incubated under dark and light conditions. The line in the box is the median. Whiskers represent the 10th and 90th percentiles. Black circles beyond the boxplots show the 5th to 95th percentiles. Different alphabets above the boxes indicate significant difference (Bonferroni post-hoc test for survival rate). Sample sizes are shown on the columns or below the boxplots.

Egg mass, hatching success, incubation period and hatchling traits differed significantly between species (Table 1). Generally, daylight fluorescent light exposure during embryonic development significantly affected incubation period and hatchling size including snout-vent length (SVL) and body mass (BM), but did not affect hatching success and hatchling survival and growth (Table 1). Post-hoc comparisons showed that light exposure did not have a significant impact on hatching success in any of the four species (Fig. 2a), but did significantly shorten incubation periods of all species (Fig. 2b). Fluorescent light exposure increased embryonic heart rates in the Chinese skink, but not in other species (Fig. 2c). In the Chinese skink, light stimulation decreased hatchling size (SVL and BM), but not head length (HL) (Fig. 3). In addition, light stimulation during embryonic development negatively affected the 30-day survival rate (Fig. 4a), but not the growth rate of skink hatchlings (Fig. 4b,c). Light stimulation had little impact, by contrast, on hatchling morphology, post-hatching survival and growth in other species (Fig. 4).

## Discussion

Currently, little is known about the effects of light on embryonic development in reptiles^12^. This study indicates that light exposure during embryonic development significantly accelerates the developmental rate of lizard embryos. Meanwhile, light stimulation negatively influenced hatchling phenotypes in skinks, but not in lacterids and geckos, probably due to the difference in eggshell thickness between species.

Light stimulates embryonic development in various lineages of oviparous animals, from invertebrates to birds^2^,^4^,^6^. Our study provides evidence of a similar effect in reptiles. There are two potential mechanisms underlying this effect. First, light may accelerate developmental rates by increasing the metabolic rates of embryos^2^,^3^,^13^,^14^,^15^. The increase of embryonic heart rates (an important index of metabolism^16^) at light conditions suggests that this mechanism may work in the skink. Second, there is evidence that patterned visual stimulation prior to hatching shortens the incubation period of leopard geckos (*Eublepharis macularius*), which suggests that light triggers egg hatching in reptiles^12^. Given that the lacertid and geckos did not increase embryonic heart rates at light condition, it will be of interest to investigate whether these species shorten their incubation period via the second mechanism.

Interestingly, the effects of light exposure during incubation on hatchling phenotypes differ among the studied lizard species. Light stimulation negatively influenced hatchling quality (e.g., smaller body size and lower survival rate of hatchlings) in the skink, but did not affect hatchling phenotypes in the lacertid and gecko. This interspecific discrepancy may reflect the differences in thickness and light transmittance of eggshells, because embryos with thin eggshells in the skink were sensitive to the light, whereas those with thick eggshells in the lacertid and gecko were not. The impact of light intensity on embryonic development and offspring phenotype also suggests the importance of eggshell thickness in protecting embryos against sunlight damage indirectly, because higher light intensity stimulated embryos to develop more rapidly, but caused more damage to offspring phenotypes in skinks (Figs 2b, 3 and 4a). Nonetheless, further studies on a wide range of reptile species are warranted, which would help us to draw a more solid conclusion on how eggshell thickness affects light transmittance and therefore embryonic development. In addition, we cannot exclude the potential contribution of the difference in the egg-laying mode of these species. The skink and lacertid lay eggs in underground nests where embryos are sheltered from light, whereas the two geckos lay eggs on walls or in the crevices of old buildings where embryos are exposed to light^10^. Due to the dark conditions of the underground nest, the skink and lacertid embryos might have lost the protective physiological mechanism over evolutionary time periods against sunlight radiation, as seen in some mammals. For example, light exposure produces more reactive oxygen species in zygotes and have detrimental effects on embryonic development in mice and hamsters^17^,^18^. Then, the lacertid embryos may develop thick eggshells to protect themselves against sunlight damage, but the skink embryos do not. In contrast, in addition to thick eggshells, gecko embryos may develop some form of protective physiological mechanism (e.g., melanin and photolyase) in response to sunlight exposure, as seen in other oviparous animals^19^,^20^. Therefore, it would be of great interest to identify if gecko embryos have developed these physiological mechanisms in future studies. Such studies will undoubtedly improve our current understanding of the mechanisms underlying the response of embryos to light.

The embryos of many oviparous vertebrates (e.g. fish, amphibians, birds) are exposed to natural light to some degree, which has led to the development of certain physiological and biochemical mechanisms (e.g. melanin and photolyase) against sunlight radiation^19^,^21^. In contrast, mammalian embryos develop under dark conditions inside the mother’s abdomen, and may have lost the mechanism to protect themselves from sunlight radiation over evolutionary time periods^17^. Given the diversity of light conditions that reptile embryos face during development, exploring the response of reptile embryos to light offers a unique opportunity to understand the evolution of light sensitivity in animal embryos. Our study suggests that some lizard embryos are sensitive to light, probably due to the evolutionary loss of the protective mechanism against sunlight radiation. A more complete comparison on how embryos respond to light conditions between species (lineages) laying eggs at dark *vs* light conditions is needed to verify this hypothesis, and can deepen our understanding of the effects of embryonic light stimulation in reptiles.

## Methods

### Ethic statements

This research was performed in accordance with the NIH *Guide for the Principles of Animal Care*. The protocol and study were approved by the Animal Ethics Committee at the Institute of Zoology, Chinese Academy of Sciences (Permit Number: IOZ14001).

### Study species

The Chinese skink (*Plestiodon chinensis*) is a medium-sized terrestrial lizard (adult snout-vent length [SVL] > 88 mm) found primarily in southern China and Vietnam^10^. During the reproductive season (May to July), adult females lay a single clutch (clutch size ranges from 9 to 25) in underground nests^22^. The northern grass lizard, *Takydromus septentrionalis*, is a small (adult SVL > 56 mm) oviparous lizard distributed in central and southern China^10^. Female northern grass lizard produce multiple clutches (clutch size ranges from 1 to 5) from early April to later July^23^. The oriental leaf-toed gecko (*Hemidactylus bowringii*) (adult SVL > 46 mm) and the Japanese gecko (*Gekko japonicus*) (adult SVL > 51 mm) are small-bodied geckos^10^,^24^,^25^. The oriental leaf-toed gecko is widely distributed in southeastern Asia, while the Japanese gecko is most common in eastern China and Japan^10^,^24^. During the reproductive season (May to August), the female geckos lay multiple clutches, with each clutch typically containing two eggs^24^,^25^.

### Animal collection and husbandry

From late April to May of 2014, we captured adult lizards of the Chinese skink, the northern grass lizard and the Japanese gecko from Wenzhou, Zhejiang province of eastern China. The oriental leaf-toed gecko was captured from Fuzhou, Fujian province of eastern China in May 2015. Lizards were captured by hand or by noose. We measured the SVL (±0.01 mm), and body mass (BM; ±0.001 g) of each captured lizard. In the laboratory, female skinks and lacertids were housed in terraria (310 × 210 × 180 mm) filled with 50 mm of moist sand. Geckos were housed in mesh cages (600 × 150** × **200 mm), the bottom of which was lined with several folds of paper to supply shelter for the animals. Each terrarium or cage contained 3–4 females and 1–2 males, and was kept in a room with a constant temperature of 23 ± 1°C and a light cycle of 12 L: 12D (0630 on and 1830 off). Supplemental heating was provided for basking from 0800 to 1600. Water and diet comprising mealworms and crickets dusted with multivitamins and minerals were provided *ad libitum*. Once they exhibited large oviductal eggs, females were moved to individual small plastic cages for egg laying. We checked these cages twice per day (at around 09:00 and 18:00) for freshly laid eggs. The collected eggs were used for the determination of eggshell thickness and light transmittance, and incubation experiments.

### Eggshell thickness and light transmittance

A total of 17, 17, 16 and 21 freshly-laid eggs (one egg from each clutch) were used to determine the thickness and light transmittance of eggshells in the Chinese skink, the northern grass lizard, the oriental leaf-toed gecko, and the Japanese gecko, respectively. The eggs were dissected and the eggshells were rinsed in distilled water and dried by blotting with a paper towel. We measured the thickness of eggshells twice to 0.001 mm using a micrometer (Mitutoyo, Japan). The mean value of the two measurements was used as eggshell thickness. We then determined the light transmittance of eggshell using a luxmeter (TES 1339 Light Meter Pro, Taiwan). The luxmeter was set up inside a black box (130 × 100 × 50 mm) with a 5-mm-diameter hole, which was exposed to the low-intensity or high-intensity lights. The low-intensity light was created by six daylight fluorescent light tubes (PHILIPS, TLD18W/865, GEER Inc, Guangdong, China) that were hung 500 mm directly above the box, while the high-intensity light was created by ten daylight fluorescent light tubes that were hung 350 mm directly above the box. The wavelength of the fluorescent light ranged from 400 to 700 nm. The light intensity inside the black box was measured when the 5-mm-diameter hole was covered with lizard eggshell or not, and the light transmittance of eggshell was calculated as the percentage of light intensity with eggshell relative to that without eggshell.

### Egg incubation and hatchling testing

The mean clutch size was 12.8 for the Chinese skink, and 3.2 for the northern grass lizard. We weighed each egg (±0.001 g) and then distributed the eggs from each clutch evenly among three incubation treatments. Female oriental leaf-toed geckos produced two eggs per clutch, which was assigned to the three treatments randomly. Female Japanese geckos produced two eggs per clutch, and the two eggs always adhered to one another. We therefore weighed the two eggs together (±0.001 g) and assigned them both to one of three incubation treatments. We allocated 47, 51, and 45 Chinese skink eggs from 14 clutches, 27, 26, 27 northern grass lizard eggs from 26 clutches, 27, 27, 26 oriental leaf-toed gecko eggs from 62 clutches, and 34, 40, and 40 Japanese gecko eggs from 65 clutches to dark, low-intensity light, and high-intensity light incubation treatments, respectively. Eggs were half-buried inside a container filled with moist vermiculite (−220 kPa), and then incubated at 28 °C in three incubators (KB400, Binder Inc. German) subject to different lighting conditions. The setup of low-intensity and high-intensity light treatments is the same as that we used to determine the light transmittance of eggshell. Light availability at the outside surface of the eggs was measured with a TES 1339 luxmeter. Luminance on the egg surfaces ranged from 752 to 985 lx for the low-intensity treatment, and from 1764 to 1970 lx for the high-intensity treatment. In contrast, eggs subjected to the dark incubation treatment (the control) received no light radiation.

To determine how light exposure affected the heart rate of lizard embryos, we subjected an additional 39 Chinese skink eggs from 4 clutches (13 eggs for each treatment), 35 northern grass lizard eggs from 20 clutches (11–12 eggs for each treatment), 21 oriental leaf-toed gecko eggs from 21 clutches (6–8 eggs for each treatment), and 22 Japanese gecko eggs from 16 clutches (7–8 eggs for each treatment) to the three incubation treatments. The heart rate of eggs were determined at the incubator whilst they were in their incubator. We measured the heart rates of the embryos halfway through the incubation period (on day 17 of incubation for skinks, day 20 for lacertids, and day 32 for geckos) using a digital egg monitor (Buddy, UK) (see Du *et al*.^26^, for detailed methodology of measuring heart rate). We recorded the heart rate of each egg every minute over a period of 10 min. The mean value of the 10 records was used as the embryonic heart rate.

Twenty days after incubation in skinks and lacertids or forty five days in geckos, we checked for new hatchlings three times per day (at around 09:00, 13:00 and 19:00). Once hatched, the hatchlings were measured for SVL, head length (HL) to 0.01 mm, and BM to 0.001 g.

### Quantifying post-hatching sizes

The skink and lacertid hatchlings were randomly allocated to terraria (400 × 300 × 200 mm, 20 mm substrate), and gecko hatchlings to mesh cages (600 × 150 × 200 mm). Terraria and cages were kept in a temperature controlled room at 28 ± 0.5 °C with a photoperiod of 12 L: 12D (from 0700 to 1900). Supplemental heating was provided for basking from 0800 to 1600. Water and a diet of mealworms and crickets dusted with multivitamins and minerals were provided *ad libitum*. All hatchlings were weighed and measured for SVL and BM again at 30 days after hatching.

### Statistical analyses

One-way or repeated-measures ANOVAs were used to determine the between-species difference in the thickness or light transmittance of eggshell. We used generalized linear mixed model to compare hatching success and hatchling survival (until 30 days of age) among incubation treatments and species, with mother identity as the random factor. Bonferroni tests were used for multiple comparisons. To avoid pseudoreplication, all analyses used clutch means for initial egg mass, incubation period and hatchling traits. Two-way ANOVAs or ANCOVAs were used to compare initial egg mass, embryonic heart rate, incubation period, hatchling morphology and growth rate across incubation treatment and species. Tukey’s tests were used for *post hoc* multiple comparisons among the treatments and species. Prior to performing the analyses of variance, we checked the raw data for normality and homogeneity of variances using Kolmogorov–Smirnov and Levene’s tests. Data were expressed as means ± SE. and statistical significance was set at α = 0.05

## Additional Information

**How to cite this article**: Zhang, Y.-P. *et al*. The effects of light exposure during incubation on embryonic development and hatchling traits in lizards. *Sci. Rep.* **6**, 38527; doi: 10.1038/srep38527 (2016).

**Publisher's note:** Springer Nature remains neutral with regard to jurisdictional claims in published maps and institutional affiliations.

## Figures and Tables

**Table 1:** Summary of two-way ANOVAs or ANCOVAs results testing the effects of light condition on embryonic development and hatchling traits across incubation treatment and species in four lizard species.

Variables	Species	Light	Species*Light
Initial egg mass (g)	***F***_**3,201**_ = **1017.07,** ***P*** < **0.001**	*F*_2,201_ = 1.29, *P* = 0.28	*F*_6,201_ = 0.04, *P* = 1.00
Heart rates (beats/min)	***F***_**3,72**_ = **39.10,** ***P*** < **0.001**	***F***_**2,72**_ = **6.31,** ***P*** = **0.003**	***F***_**6,72**_ = **3.59,** ***P*** = **0.004**
Hatching success (%)	***F***_**3,405**_ = **4.819,** ***P*** = **0.003**	*F*_2,405_ = 0.08, *P* = 0.92	*F*_6,405_ = 0.52, *P* = 0.79
Incubation period (days)	***F***_**3,201**_ = **9284.56,** ***P*** < **0.001**	***F***_**2,201**_ = **135.84,** ***P*** < **0.001**	***F***_**6,201**_ = **21.24,** ***P*** < **0.001**
Snout-vent length (mm)	***F***_**3,200**_ = **193.27,** ***P*** < **0.001**	***F***_**2,200**_ = **3.69,** ***P*** = **0.03**	***F***_**6,200**_ = **2.18,** ***P*** = **0.05**
Body mass (g)	***F***_**3,200**_ = **397.97,** ***P*** < **0.001**	***F***_**2,200**_ = **6.27,** ***P*** = **0.002**	***F***_**6,200**_ = **3.16,** ***P*** = **0.006**
Head length (mm)	***F***_**3,200**_ = **65.11,** ***P*** < **0.001**	*F*_2,200_ = 0.44, *P* = 0.65	***F***_**6,200**_ = **2.54,** ***P*** = **0.02**
Survival rate (%)	***F***_**3,343**_ = **9.17,** ***P*** < **0.001**	*F*_2,343_ = 1.23, *P* = 0.29	*F*_6,343_ = 1.90, *P* = 0.08
Growth rate of SVL (mm/day)	***F***_**3,157**_ = **22.99,** ***P*** < **0.001**	*F*_2,157_ = 1.86, *P* = 0.16	*F*_6,157_ = 0.48, *P* = 0.82
Growth rate of BM (g/day)	***F***_**3,157**_ = **54.03,** ***P*** < **0.001**	*F*_2,157_ = 2.22, *P* = 0.11	*F*_6,157_ = 0.47, *P* = 0.83

Significant differences are indicated in bold.
